# Neuroactive Phytochemicals as Multi-Target Modulators of Mental Health and Cognitive Function: An Integrative Review

**DOI:** 10.3390/ijms26188907

**Published:** 2025-09-12

**Authors:** Halina Tkaczenko, Lyudmyla Buyun, Renata Kołodziejska, Piotr Kamiński, Natalia Kurhaluk

**Affiliations:** 1 Institute of Biology, Pomeranian University in Słupsk, Arciszewski St. 22B, 76-200 Słupsk, Poland; halina.tkaczenko@upsl.edu.pl; 2 Department of Tropical and Subtropical Plants, M.M. Hryshko National Botanic Garden, National Academy of Science of Ukraine, 01003 Kyiv, Ukraine; orchids.lyuda@gmail.com; 3 Department of Medical Biology and Biochemistry, Collegium Medicum in Bydgoszcz, Nicolaus Copernicus University in Toruń, M. Karłowicz St. 24, 85-092 Bydgoszcz, Poland; renatak@cm.umk.pl; 4 Department of Medical Biology and Biochemistry, Division of Ecology and Environmental Protection, Collegium Medicum in Bydgoszcz, Nicolaus Copernicus University in Toruń, M. Skłodowska-Curie St. 9, 85-094 Bydgoszcz, Poland; piotr.kaminski@cm.umk.pl; 5 Department of Biotechnology, Institute of Biological Sciences, Faculty of Biological Sciences, University of Zielona Góra, Prof. Z. Szafran St. 1, 65-516 Zielona Góra, Poland

**Keywords:** phytochemicals, oxidative stress, neuroinflammation, gut–brain axis, cognitive function, mental health, synaptic plasticity, natural compounds, neuroprotection

## Abstract

The growing prevalence of mental health issues and cognitive impairment poses a significant challenge to global public health. Conditions such as depression, anxiety, neurodegenerative diseases, and stress-related cognitive dysfunction are becoming more common, while conventional pharmacotherapies are often limited by suboptimal efficacy, adverse side effects, and concerns about long-term use. Against this backdrop, neurophytochemistry—the study of plant-derived bioactive compounds—has emerged as a promising area of research. This review explores the potential of selected phytochemicals to support mental well-being and cognitive function via various molecular mechanisms. Compounds such as apigenin, hesperidin, and epigallocatechin gallate have been shown to have a significant impact on key regulatory pathways. These include enhancing neurogenesis via brain-derived neurotrophic factor, modulating neurotransmitter systems (such as GABA and serotonin), and attenuating oxidative stress and neuroinflammation. The therapeutic relevance of these compounds is discussed in the context of depression, anxiety, Alzheimer’s disease, Parkinson’s disease, and stress-related cognitive dysfunction, often referred to as ‘brain fog’. This review synthesizes evidence published between 2010 and 2025 from several scientific databases, including PubMed, Scopus, Web of Science, and Embase. Preliminary evidence from in vitro studies and animal models indicates that neurophytochemicals could enhance synaptic plasticity, protect neurons from oxidative damage, and modulate inflammatory pathways, particularly those involving NF-κB and the Nrf2/ARE antioxidant response. In addition, early human clinical trials have shown that phytochemical supplementation can lead to improvements in mood regulation, stress response, and cognitive performance. Furthermore, emerging evidence suggests that the gut–brain axis plays a key role in mediating the effects of phytochemicals. Several compounds have been found to modulate the composition of gut microbiota in ways that could enhance the function of the central nervous system. While the initial results are encouraging, more high-quality clinical trials and mechanistic studies are required to validate these findings, optimize dosage regimens, and guarantee the safety and efficacy of long-term use. Thus, neurophytochemicals represent a promising integrative approach to alleviating the increasing burden of mental and cognitive disorders through naturally derived therapeutic strategies.

## 1. Introduction

In recent years, the global burden of mental health conditions and cognitive decline has increased significantly, posing a major public health concern [1]. Depression affects over 300 million people worldwide, according to the World Health Organization (WHO), and is identified as the leading cause of disability worldwide [2]. At the same time, neurodegenerative diseases such as Alzheimer’s are increasing at an unprecedented rate [3]. Multiple reviews have outlined that aging is the primary risk factor for developing neurodegenerative diseases and their associated comorbidities, including vascular, metabolic, and immune diseases in the elderly [4]. As global life expectancy continues to rise, the prevalence of these diseases is expected to increase, placing a significant burden on healthcare systems worldwide. Unfortunately, current therapeutic options are very limited, with no interventions capable of halting or reversing the progression of these diseases [5].

Studies have emphasized the significance of modifiable lifestyle factors, particularly diet, in relation to mental and cognitive health [6]. Plant-derived compounds possess essential antioxidant, anti-inflammatory, anti-cholinesterase, and anti-apoptotic properties for maintaining nervous system integrity [7]. Unlike synthetic drugs that act on a single target, phytochemicals have a variety of effects by modulating neurotransmitter systems, alleviating neuroinflammation, stimulating neurogenesis, and protecting against oxidative stress. This makes them promising agents for preventing and treating mental disorders [7].

Recent insights from the emerging field of nutritional psychiatry highlight the growing therapeutic potential of dietary phytochemicals in clinical settings. A recent cross-sectional analysis of NHANES 2017–2018 data revealed a significant inverse association between dietary flavonoid intake and anxiety symptoms in adults [8]. This finding is supported by observational studies of large cohorts showing that higher ‘flavodiet’ scores are associated with a lower incidence of dementia and improved mental health outcomes in aging populations [9]. Furthermore, a systematic review of nutraceuticals suggests that diets rich in fiber, omega-3 fatty acids, and phytochemicals have neuroprotective and anti-inflammatory properties that can benefit people with psychiatric conditions [10]. A 2025 study identified plant-derived polyphenols as promising adjunctive agents in the management of schizophrenia, highlighting their potential applications beyond mood disorders [11].

Polyphenols, flavonoids, terpenoids, and alkaloids, which are widely distributed among different plant species, have demonstrated significant neuroprotective effects through their antioxidant, anti-inflammatory, and neuromodulatory properties [12,13]. Several authors have argued that recent advances in analytical methods have facilitated a deeper understanding of the interactions between these bioactive compounds and specific neuronal receptors and signaling cascades. For instance, flavonoids such as quercetin and kaempferol have been shown to inhibit monoamine oxidase (MAO) activity, thereby modulating monoaminergic neurotransmission. This is an essential mechanism that is often dysregulated in mood and anxiety disorders [14,15]. This research highlights the specific molecular mechanisms by which neuroactive phytochemicals exert their effects. For instance, apigenin and hesperidin have been shown to increase levels of brain-derived neurotrophic factor (BDNF), a key mediator of synaptic plasticity and memory formation [16,17]. Additionally, epigallocatechin gallate (EGCG), a major catechin found in green tea, modulates GABAergic and dopaminergic neurotransmission systems while acting as a potent antioxidant [18]. These phytochemicals’ multiple actions fit well with the complex, multifactorial pathophysiology of mental disorders, making them promising candidates for complementary or alternative therapeutic approaches, as reflected in the growing body of peer-reviewed publications spanning molecular biology, pharmacology, and clinical neuroscience [19].

Furthermore, evidence from clinical trials and meta-analyses suggests that certain phytochemicals have therapeutic potential in alleviating the symptoms of major depressive disorder (MDD) [20], generalized anxiety disorder (GAD) [21], and mild cognitive impairment (MCI) [22]. For instance, saffron (*Crocus sativus* L.) extracts standardized for crocin and safranal content have demonstrated antidepressant efficacy similar to that of selective serotonin reuptake inhibitors (SSRIs) in certain randomized controlled trials [23,24,25]. Similarly, curcumin, the active compound in *Curcuma longa* L. (turmeric), has demonstrated efficacy in reducing depressive and anxiety symptoms, particularly when used alongside conventional pharmacological treatments [26,27].

Recent advances have further emphasized the importance of plant-derived compounds in a variety of biomedical applications. Phytochemicals are increasingly recognized for their neuroprotective potential and broad-spectrum activities, including anti-cancer, anti-inflammatory, cardioprotective, anti-diabetic, and antimicrobial effects [7,28]. For example, flavonoids such as quercetin, kaempferol, and luteolin have been shown to modulate key signaling pathways involved in oxidative stress, apoptosis, and immune regulation, thereby helping to prevent and manage disease. Their pleiotropic biological activities are important because they highlight their relevance in integrative medicine and support their inclusion in dietary strategies aimed at promoting systemic and neurological health [29,30,31]. Recent studies further expand this perspective. Rana and Mumtaz (2025) reviewed prunin, an emerging anticancer flavonoid, demonstrating its ability to counteract oxidative stress, modulate inflammatory signaling, and act as an immunomodulatory adjuvant to enhance the efficacy of immune checkpoint inhibitors in preclinical cancer models [32]. Similarly, isorhamnetin has been reported to exert potent anti-metastatic effects by suppressing PI3K/AKT and STAT3 signaling, downregulating VEGF expression, and stabilizing endothelial barriers, thereby inhibiting angiogenesis and cancer cell dissemination [33].

This article is an example of a narrative review, which, by its very nature, seeks to consolidate and critically examine the most influential studies on neuroactive phytochemicals from 2010 to 2025. Analyzing data from prominent scientific databases such as PubMed, Scopus, Web of Science, and Embase helped to identify trends, highlight knowledge gaps, and propose new research directions. Particular emphasis was placed on human studies, in vitro models, and animal behavioral paradigms to ensure translational relevance. Particular attention was also paid to the methodological quality of the included studies, focusing on the standardization of phytochemicals, dosage ranges, the duration of interventions, and the use of validated psychometric instruments. The heterogeneity of study designs and outcome measures was critically assessed to provide a nuanced understanding of the efficacy and limitations of neurophytochemicals in different populations.

By presenting neurophytochemicals as a conceptual bridge between nutrition and mental health, the review contributes to the growing body of evidence supporting lifestyle interventions in psychiatry. Additionally, it highlights the potential of phytochemicals as innovative tools for precision mental healthcare and functional food development, in line with contemporary personalized medicine principles. By synthesizing current knowledge from various scientific disciplines, the review aims to promote a more comprehensive understanding of how dietary phytochemicals influence neural function and mental well-being. The findings are expected to inform future research directions, support the development of clinical applications, and guide public health strategies that promote mental resilience through natural, plant-based interventions.

## 2. Key Neuroactive Phytochemicals and Their Dietary Sources

**Figure 1:**
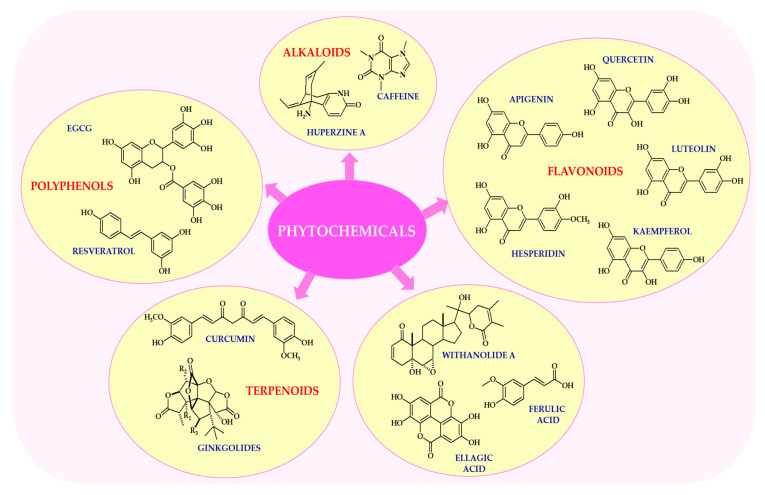
Chemical structure of selected neuroactive phytochemicals.

Neurophytochemicals are a diverse group of biologically active plant compounds with neuroprotective and psychotropic properties (Figure 1). The most extensively studied of these are flavonoids, alkaloids, terpenoids, and polyphenols. These compounds have been shown to have a number of beneficial effects on brain function, primarily due to their antioxidant, anti-inflammatory, and neuromodulatory properties [12,13].

As outlined in multiple reviews, the field has increasingly recognized that flavonoids are a large subclass of polyphenolic compounds widely distributed in fruits, vegetables, and plant-based beverages such as tea and wine, as reported by Dias et al. [28]. For example, quercetin, which is found in apples, onions, and berries, has potent antioxidant and anti-inflammatory properties, and has been shown to modulate neuronal signaling pathways [29]. Luteolin, found in celery, parsley, and chamomile tea, has neuroprotective effects by attenuating microglial activation and reducing neuroinflammation, demonstrating a consistent pattern across various contexts [30]. Kaempferol, which is abundant in kale, broccoli, and green tea, supports mitochondrial function and exhibits neurotrophic activity [31]. These flavonoids exert their neuroactive effects by modulating intracellular signaling cascades, including the ERK, PI3K/Akt, and NF-κB pathways [29,30,31].

Alkaloids are nitrogen-containing compounds with diverse pharmacological properties [34]. One notable finding from the literature is that caffeine, the most widely consumed psychoactive alkaloid, is found naturally in coffee, tea, and cocoa. It acts as an adenosine receptor antagonist, thereby enhancing alertness, cognitive performance, and mood [35]. Numerous studies have highlighted the importance of huperzine A, which is extracted from *Huperzia serrata* (Thunb.) Trevis. and has been shown to improve memory and learning due to its potent acetylcholinesterase inhibitory properties. It is currently used as an adjunctive therapy in the treatment of Alzheimer’s disease [36,37]. It is important to note that alkaloids often exhibit dose-dependent effects, and their safety profile requires careful consideration, particularly in vulnerable populations [34].

Terpenoids are a structurally diverse class of natural compounds derived from isoprene units, which can serve as the basis for future theoretical and empirical work [38]. Ginkgolides, which are isolated from the leaves of *Ginkgo biloba* L., have been shown to improve cerebral blood flow, protect neurons from ischemic damage, and reduce oxidative stress [39]. Curcumin, the main curcuminoid found in turmeric, has potent anti-inflammatory and antioxidant properties. It also modulates the aggregation of amyloid-β and has been extensively investigated as a potential treatment for neurodegenerative diseases such as Alzheimer’s and Parkinson’s [40,41]. However, its limited bioavailability remains a major challenge, prompting the development of advanced delivery systems, including nanoparticle carriers and lipid-based formulations [42].

Polyphenols, particularly stilbenes and catechins, are well known for their ability to modulate brain function, demonstrating a consistent pattern across various contexts [43]. As reported by several independent research groups, resveratrol, which is found in grapes, red wine, and peanuts, activates sirtuin pathways and promotes mitochondrial biogenesis, thereby contributing to neuroprotection and extending lifespan [44,45]. It has been demonstrated that EGCG exhibits anxiolytic and antidepressant properties, primarily by modulating GABAergic and dopaminergic neurotransmission systems [46]. Furthermore, polyphenols have been shown to promote neurogenesis and enhance synaptic plasticity by upregulating BDNF expression [47].

A closer examination of the literature over the past decade reveals a consistent emphasis on phytochemicals, particularly in studies showing that incorporating neuroactive phytochemicals into the diet can be effectively achieved through the regular consumption of a variety of plant-based foods [48]. Rich sources of neuroprotective phytochemicals include fruits (such as berries, apples, and grapes), vegetables (such as broccoli, spinach, and celery), nuts, seeds, teas (including green, black, and herbal varieties), and spices (e.g. turmeric), as reflected in the growing body of peer-reviewed publications spanning molecular biology, pharmacology, and clinical neuroscience [49,50]. Although there are currently no universal dietary guidelines specifying optimal intake levels for individual phytochemicals, a diverse and balanced diet rich in colorful plant foods—following models such as the Mediterranean or MIND diets—has been consistently associated with improved cognitive health outcomes [51,52]. Supplementation may be appropriate in cases of dietary inadequacy or specific therapeutic needs, but should be undertaken within evidence-based guidelines and under clinical supervision (Table 1).

A growing body of recent research has highlighted the therapeutic potential of neurophytochemicals, which are plant-derived compounds that exert biological effects on the nervous system. There is growing interest in using them to treat and prevent neurological disorders, particularly given their diverse mechanisms of action within the central nervous system (CNS) [68]. These compounds can modulate a variety of physiological and molecular pathways, making them promising candidates for adjunctive therapies in neurodegenerative, psychiatric, and neuroinflammatory conditions [69]. Furthermore, phytochemicals often have a favorable safety profile compared to conventional pharmacological agents, exhibiting lower toxicity and fewer side effects, which increases their appeal as complementary or standalone therapeutic options [7].

Recent advances in phytoneurology have demonstrated that plant-derived compounds can play an important role in treating nervous system disorders. These natural products can act on neurons at the molecular level to support repair mechanisms and protect against various forms of damage [70]. One well-documented example is *Bacopa monnieri* (L.) Wettst., a key herb in Ayurvedic medicine that contains bacosides, which have been shown to activate the MAPK/ERK signaling pathway [71,72]. This pathway has been associated with memory enhancement and neuronal survival [73]. Another extensively studied botanical is *Ginkgo biloba*, which is renowned for its neuroprotective properties, primarily due to its flavonoid content [39]. These flavonoids modulate key signaling pathways, such as PI3K/Akt and MAPK, thereby promoting neuronal viability [74]. Additionally, compounds such as rosmarinic acid (from *Salvia rosmarinus* Spenn.) and salidroside (from *Rhodiola rosea* L.) have demonstrated encouraging neurotrophic and anti-apoptotic properties, thereby enhancing neuronal resilience in stressful conditions [75,76,77,78].

**Figure 2:**
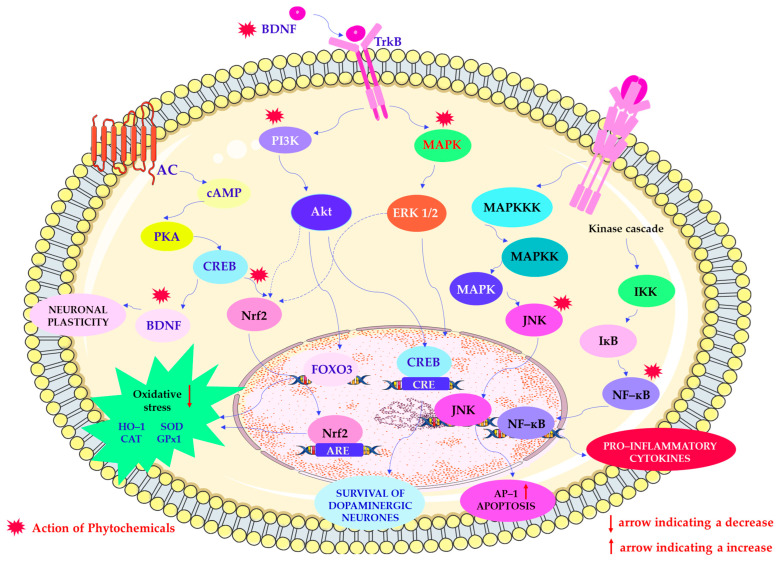
Molecular mechanism of action of neuroactive phytochemicals. Phytochemicals improve neurological function by modulating pro-inflammatory and oxidative molecular pathways. They activate the Nrf2/ARE pathway, leading to increased expression of antioxidant enzymes (HO-1, SOD, CAT, and GPx), and promote neuronal viability by modulating the PI3K/Akt and MAPK signaling pathways. Phytochemicals can also increase BDNF levels. BDNF plays a key role in differentiation, neuronal growth, neurogenesis, modulation of plasticity, and neurodegeneration. They inhibit inflammation by modulating JNK, AMPK, and NF-κB pathways. Abbreviations: AC—Adenylate cyclase; Akt—Protein kinase B (PKB); AMPK—AMP-activated protein kinase; AP-1—Activator protein 1; ARE—Antioxidant response element; BDNF—Brain-derived neurotrophic factor; CAT—Catalase; cAMP—Cyclic adenosine monophosphate; CRE—cAMP response elements; CREB—Transcription factor capable of binding DNA; ERK 1/2—Extracellular signal-regulated kinases; FOXO3—Forkhead family of transcription factor; GPx—glutathione peroxidase; HO-1—Heme oxygenase; IKK—IκB kinase; IκB—Inhibitor of nuclear factor kappa B; JNK—c-Jun N-terminal kinase; MAPK/MAPKK/MAPKKK—Mitogen-activated protein kinases and their upstream kinases; NF-κB—Nuclear Factor-kappa B; Nrf2—Transcription Factor 2; PI3K—Phosphatidylinositol 3-kinase; PKA—Protein kinase A; SOD—Superoxide dismutase; TrkB—Tropomyosin receptor kinase B. This Figure was created using Servier Medical Art (available at https://smart.servier.com/) (accessed on 1 May 2025).

An important mechanism currently under investigation is the influence of phytochemicals on neurogenesis and synaptic plasticity. Studies have shown that certain phytochemicals can increase levels of BDNF (a protein critical for the proliferation of new neurons and the consolidation of synaptic connections), offering promising therapeutic potential, particularly in the context of neurodegenerative diseases [79]. This is highly relevant to conditions such as depression, Alzheimer’s disease, and Parkinson’s disease, where neurogenesis and synaptic remodeling are often impaired [80,81,82]. The formation of new neurons and the remodeling of synaptic connections are essential for maintaining cognitive resilience and functional recovery [83]. Therefore, compounds found in *Withania somnifera* (L.) Dunal (ashwagandha), particularly withanolides, have been shown to activate transcription factors such as CREB and FOXO3a. This leads to increased BDNF expression and enhanced neuronal growth and plasticity, particularly in the hippocampus [84,85]. Similarly, turmeric exerts neurotrophic effects through its active component, curcumin, which upregulates BDNF via CREB activation, thereby supporting both neurogenesis and long-term potentiation [86,87]. Ginseng (*Panax ginseng* C.A. Meyer) has also been shown to activate the ERK and PI3K/Akt pathways, which enhance BDNF-mediated neuroplasticity [88,89]. Furthermore, recent studies have highlighted the neuroregenerative potential of *Centella asiatica* (L.) Urb. (gotu kola), which is rich in triterpenoids such as asiaticoside and promotes neurite outgrowth and synaptic connectivity by modulating key neurodevelopmental pathways [90]. The molecular mechanism of action of neuroactive phytochemicals is shown in Figure 2.

Another important area of interest is the regulation of neurotransmitter systems, particularly those involving serotonin, dopamine, and GABA. However, as noted in the aforementioned reviews [91,92], findings remain somewhat heterogeneous depending on the disease model and compound studied. Phytochemicals have been shown to influence the balance and activity of these neurotransmitters, thereby contributing to mood stabilization, improved cognitive performance, and the alleviation of anxiety-related symptoms [91,92]. Consequently, plant-derived compounds such as curcumin, resveratrol, and flavonoids are attracting increasing interest for their potential antidepressant and anxiolytic properties [93,94,95]. Equally important is the broader role of phytochemicals in supporting neurotransmitter systems involved in emotional and mental regulation [92]. Proper neurotransmitter signaling is fundamental to maintaining mood balance, cognitive function, and healthy sleep patterns [96]. One notable example is *Mucuna pruriens* (L.) DC, a natural source of L-DOPA, which increases dopamine availability in the brain, making it a valuable adjunct in the treatment of Parkinson’s disease [97]. Additionally, alkaloids in *M. pruriens* have been reported to inhibit monoamine oxidase B (MAO-B), thereby prolonging dopamine activity by reducing its enzymatic degradation [98].

Numerous studies have highlighted the importance of neurophytochemicals, which have the ability to influence the balance and activity of neurotransmitters. This helps to stabilize mood, improve cognitive function, and reduce anxiety symptoms. *Hypericum perforatum* L. (St. John’s wort) is a notable example of this phenomenon, exerting its effects by inhibiting the reuptake of serotonin, dopamine, and norepinephrine via the blocking of synaptic transporters, in a manner analogous to certain conventional antidepressants [99]. Additionally, *Valeriana officinalis* L. (valerian root) has been shown to enhance GABAergic signaling by modulating GABA-A receptor activity, thereby promoting relaxation and anxiolytic effects [100]. *Passiflora incarnata* L. (passionflower) and *Lavandula latifolia* Medic. (lavender) extracts have also demonstrated GABAergic and serotonergic modulation, indicating their potential inclusion in therapies for generalized anxiety and stress-related disorders [101,102,103].

Given the central role of oxidative stress and neuroinflammation in the pathogenesis of many neurological disorders, the anti-inflammatory and antioxidant properties of neurophytochemicals represent a key area of investigation [104]. By neutralizing free radicals and modulating inflammatory responses, phytochemicals have the potential to exert neuroprotective effects and slow the progression of neurodegenerative diseases [105]. For instance, resveratrol, which is found in high concentrations in the skins of grapes (*Vitis vinifera* L.), has been demonstrated to activate the Nrf2/ARE signaling pathway, thereby increasing the expression of antioxidant enzymes such as superoxide dismutase (SOD) and catalase [106]. Conversely, resveratrol inhibits NF-κB signaling, resulting in reduced production of pro-inflammatory cytokines [107]. A similar dual effect is observed with green tea polyphenols (e.g., EGCG from *Camellia sinensis* (L.) Kuntze), which suppress oxidative stress and inflammation by modulating the JNK and AMPK pathways [108]. Other phytochemicals, such as quercetin and kaempferol, contribute to neuroprotection by modulating redox-sensitive transcription factors and downregulating key inflammatory mediators, thereby attenuating neuroinflammatory cascades [31,109].

A substantial body of research has demonstrated the particular value of phytochemicals with anti-inflammatory and antioxidant properties in the treatment of diseases associated with chronic oxidative stress [110,111]. Inflammatory responses in the brain have been shown to contribute to neuronal damage and cognitive decline [112]. Turmeric, which is rich in curcumin, activates the Nrf2 pathway, thereby increasing the expression of antioxidant enzymes [113]. Conversely, it has been observed to inhibit NF-κB, a key regulator of inflammation, thereby reducing levels of pro-inflammatory cytokines such as interleukin-6 (IL-6) and tumor necrosis factor-alpha (TNF-α) [114]. Furthermore, there is growing evidence that long-term supplementation with these bioactives may alleviate mitochondrial dysfunction and promote cellular energy homeostasis in neuronal tissue, providing an additional protective mechanism against age-related cognitive decline [61].

## 3. Plant Compounds and Blood–Brain Barrier Integrity in Brain Disorders

Recent studies have highlighted the modulation and protection of the blood–brain barrier (BBB) by phytochemicals as a promising and rapidly emerging area of research. The BBB is a selective, semi-permeable interface that separates circulating blood from the brain parenchyma and the extracellular fluid of the central nervous system (CNS), demonstrating a consistent pattern across various contexts. It plays a critical role in maintaining neural homeostasis by preventing the entry of neurotoxic substances, pathogens, and peripheral immune cells [115]. However, there is increasing evidence that BBB dysfunction is a key factor in the pathogenesis of several neurodegenerative and neuroinflammatory disorders, including Alzheimer’s disease (AD), Parkinson’s disease (PD), multiple sclerosis (MS), ischemic stroke, and epilepsy [116].

**Figure 3:**
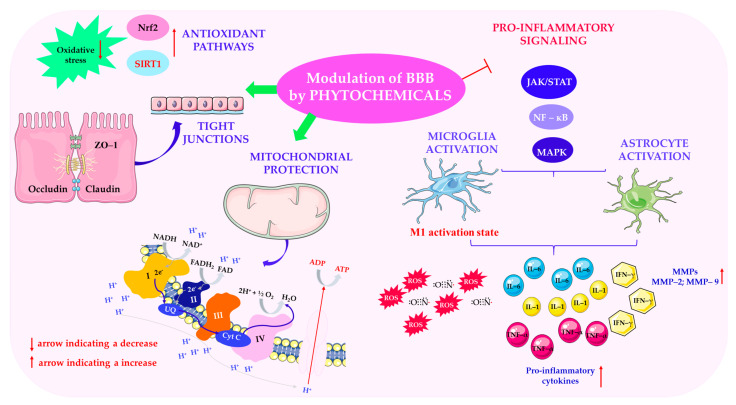
Modulation of the blood–brain barrier (BBB) by phytochemicals. Phytochemicals enhance BBB integrity by activating antioxidant pathways, upregulating tight junction proteins (e.g., ZO-1), and protecting mitochondrial function. They inhibit proinflammatory signaling pathways by reducing the activation of microglia and astrocytes and decreasing the release of proinflammatory mediators such as IL-1, IL-6, TNF-α, and INF-γ. These effects collectively preserve BBB permeability and neuronal homeostasis under conditions of stress or neuroinflammation. Abbreviations: I—NADH reductase; II—Succinate dehydrogenase; III—Cytochrome reductase; IV—Cytochrome oxidase; ADP—Adenosine diphosphate; ATP—Adenosine triphosphate; BBB—Blood–brain barrier; Cyt C—Cytochrome C; FADH_2_—Flavin adenine dinucleotide (reduced form); FAD—Flavin adenine dinucleotide (oxidized form); IL-1/6— Interleukin 1/6; INF-γ—Interferon gamma; JNK—c-Jun N-terminal kinase; MAPK—Mitogen-activated protein kinases; M1—M1 phenotype; MMPs—Matrix metalloproteinases; MMP-2/9—Matrix metalloproteinase-2/9; NADH—Nicotinamide adenine dinucleotide (reduced form); NAD^+^—Nicotinamide adenine dinucleotide (oxidized form); NF-κB—Nuclear Factor-kappa B; Nrf2—Transcription Factor 2; ROS—Reactive oxygen species; SIRT1—Silent information regulator 1; STAT—Signal transducer and activator of transcription; TNF-α—Tumor necrosis factor; UQ—Ubiquinone; ZO-1—Zonula Occludens-1. This Figure was created using Servier Medical Art (available at https://smart.servier.com/) (accessed on 1 May 2025).

In AD, BBB breakdown precedes cognitive decline and is associated with pericyte loss, reduced expression of tight junction proteins, and accumulation of neurotoxic amyloid-beta (Aβ) peptides. Disruption of the BBB facilitates the entry of circulating Aβ, inflammatory cells, and cytokines into the brain, exacerbating neuroinflammation and contributing to synaptic dysfunction [117]. Numerous studies have highlighted the importance of EGCG, which has been shown to inhibit Aβ-induced expression of matrix metalloproteinase (MMP-9), preserve levels of the tight junction proteins claudin-5 and ZO-1, and improve cognitive performance in AD mouse models [118]. The modulation of blood–brain barrier processes by phytochemicals is shown in Figure 3.

In PD, neurodegeneration in the *substantia nigra* is associated with endothelial damage induced by oxidative stress, increased BBB permeability, and α-synuclein accumulation [119]. As quercetin and resveratrol have been shown to reduce oxidative damage by activating the Nrf2/ARE antioxidant pathway and reducing levels of pro-inflammatory cytokines (e.g., TNF-α and IL-6), they could be effective in treating PD [120].

As reported by several independent research groups, multiple sclerosis is an autoimmune demyelinating disease characterized by lymphocyte infiltration into the CNS, a process largely facilitated by BBB disruption. Pro-inflammatory cytokines such as IL-1β, IFN-γ, and TNF-α induce the downregulation of tight junction proteins and increase the transendothelial migration of immune cells [121]. Luteolin has been shown to inhibit cytokine-induced barrier breakdown by modulating NF-κB and JAK/STAT signaling, thereby limiting leukocyte entry and reducing lesion volume in experimental autoimmune encephalomyelitis models [122].

In the context of ischemic stroke, hypoxia and reperfusion injury trigger the release of reactive oxygen species (ROS) and pro-inflammatory mediators. This leads to the activation of matrix metalloproteinases (MMP-2 and MMP-9), extracellular matrix degradation, and endothelial apoptosis [123]. Rosmarinic acid has been shown to exert neurovascular protection by activating AMP-activated protein kinase (AMPK), inhibiting MMP-9, and preserving the ultrastructure of the BBB [124]. It also reduces infarct size and improves neurological outcome in animal models of stroke [125].

In epilepsy, BBB dysfunction may contribute to seizure initiation and propagation by allowing albumin and other serum proteins to enter the brain parenchyma, thereby triggering astrocyte activation and neuronal hyperexcitability [126]. Curcumin has demonstrated antiepileptic activity by restoring BBB integrity, suppressing IL-1β, and increasing the expression of tight junction proteins [127].

Mechanistically, phytochemicals modulate BBB integrity through the following key pathways: (1) inhibition of matrix metalloproteinases (MMP-2 and MMP-9); (2) activation of antioxidant pathways (e.g., Nrf2/ARE, AMPK, and SIRT1); (3) suppression of pro-inflammatory signaling (e.g., NF-κB, MAPK, and JAK/STAT); (4) upregulation of tight junction proteins; and (5) protection of mitochondria and support of energy metabolism. These mechanisms are demonstrated in the works of the authors listed below. For example, the inhibition of MMP-2 and MMP-9 by phytochemicals reduces the degradation of the extracellular matrix and the breakdown of tight junctions [128,129], while the activation of antioxidant pathways (e.g., Nrf2/ARE, AMPK, and SIRT1) protects endothelial cells from oxidative stress [130]. Phytochemical-induced suppression of pro-inflammatory signaling (NF-κB, MAPK, and JAK/STAT) reduces cytokine-mediated permeability [131]. Upregulation of tight junction proteins helps to maintain BBB selectivity and homeostasis, while mitochondrial protection and improved energy metabolism support endothelial resistance and function [132,133]. Together, these mechanisms highlight the potential of phytochemicals as adjunctive therapies for neurodegenerative and neurovascular disorders. Further clinical and translational studies are needed to confirm the efficacy, pharmacokinetics, and BBB permeability of these neurophytochemicals in human populations.

## 4. Key Neuroactive Phytochemicals and Their Dietary Sources

A comparative table of major neuroactive phytochemicals, including their dietary sources, molecular mechanisms of action, neuroprotective functions, and associated neurological disorders, is presented below (Table 2).

Numerous studies have highlighted the neuroprotective properties of apigenin, a naturally occurring flavone found in plants such as chamomile (*Matricaria chamomilla* L.) and parsley (*Petroselinum crispum* (Mill.) Fuss). These effects are mainly mediated by apigenin’s modulation of neurotransmission, neuroinflammation, and oxidative stress. At a molecular level, apigenin enhances GABAergic transmission by binding to the benzodiazepine site of the GABA-A receptor, producing anxiolytic and sedative effects similar to those of conventional benzodiazepines [136]. Additionally, apigenin inhibits the NF-κB signaling pathway, resulting in the downregulation of pro-inflammatory cytokines such as TNF-α and IL-1β [137]. Apigenin also activates the Nrf2/ARE signaling cascade, thereby promoting the expression of endogenous antioxidant enzymes such as haem oxygenase-1 (HO-1) and SOD, which reduce oxidative damage in neuronal cells [138,139].

Hesperidin, a flavanone glycoside predominantly found in citrus fruits, has been shown to exert multiple neuroprotective effects [140]. One of its key mechanisms is the upregulation of BDNF via activation of the cAMP response element-binding protein (CREB) signaling pathway, which plays a critical role in neurogenesis, synaptic plasticity, and memory consolidation [141]. Furthermore, hesperidin modulates monoaminergic neurotransmission by increasing serotonin and dopamine levels through the inhibition of monoamine oxidase (MAO) activity [142]. At the inflammatory level, hesperidin suppresses the TLR4/NF-κB signaling pathway, thereby reducing microglial activation and the release of pro-inflammatory mediators [143]. Furthermore, hesperidin has been shown to enhance the integrity of the BBB by increasing the expression of tight junction proteins and protecting cerebral endothelial cells from oxidative damage [144].

Recent evidence has shown that EGCG exhibits potent antioxidant and neuroprotective activities [145]. Mechanistically, EGCG activates the Nrf2 signaling pathway, thereby enhancing the transcription of antioxidant and detoxifying enzymes, such as glutathione S-transferase (GST) and SOD [146]. Notably, EGCG inhibits the aggregation of misfolded proteins, such as amyloid β and α-synuclein, which are hallmarks of AD and PD, respectively [147,148]. Furthermore, EGCG suppresses neuroinflammation by downregulating c-Jun N-terminal kinase (JNK) and NF-κB signaling, while promoting neuronal survival by activating the PI3K/Akt and ERK1/2 pathways [149,150].

Curcumin is another compound recognized for its neuroprotective effects and broad-spectrum activity [151]. It activates the Nrf2/ARE pathway, thereby increasing the expression of antioxidant enzymes such as catalase and glutathione peroxidase (GPx) and reducing oxidative stress in neural tissue [152]. Curcumin also inhibits NF-κB activity and downregulates the expression of pro-inflammatory enzymes, including cyclooxygenase-2 (COX-2) and inducible nitric oxide synthase (iNOS) [114,153]. Curcumin also improves neuroplasticity and cognitive function by upregulating BDNF via the ERK/CREB pathway [154]. Furthermore, curcumin prevents neuronal apoptosis by modulating mitochondrial pathways, specifically by suppressing pro-apoptotic proteins (e.g., Bax) and upregulating anti-apoptotic markers such as Bcl-2 [155].

Two other prominent polyphenolic phytochemicals, quercetin and rosmarinic acid, have attracted considerable attention in preclinical and translational neuroscience research. Found in onions, apples, berries, and leafy vegetables, quercetin is able to cross the BBB and has been shown to activate sirtuin 1 (SIRT1), which is a key regulator of mitochondrial biogenesis, neuronal energy metabolism, and longevity [156,157]. It also induces the Nrf2 pathway, thereby reducing lipid peroxidation and increasing cellular antioxidant capacity [158,159]. Additionally, quercetin inhibits Toll-like receptor 4 (TLR4), which leads to suppression of microglial activation and consequent reduction in the release of neurotoxic cytokines [160].

Building on the work of Elsheikh et al. (2025), it is evident that rosmarinic acid, which is found in rosemary and lemon balm, maintains BBB integrity by upregulating tight junction proteins, such as claudin-5 and occludin [161]. Rosmarinic acid also reduces excitotoxic damage by modulating glutamatergic receptor activity, particularly NMDA receptors [162], and inhibits neuroinflammation by suppressing COX-2 and downregulating TNF-α [163,164]. 

## 5. Phytochemicals in the Treatment of Specific Mental and Cognitive Disorders

**Figure 4:**
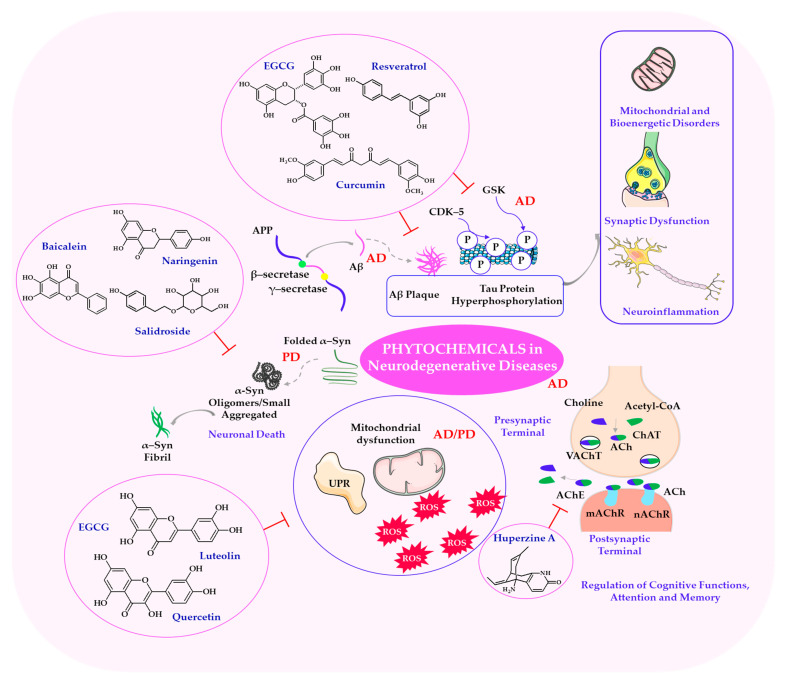
Neuroprotective effects of phytochemicals in neurodegenerative diseases. Phytochemicals exert multifaceted neuroprotection by inhibiting protein aggregation and toxicity. This includes the prevention of Aβ aggregation, the reduction in α-synuclein toxicity, and the modulation of Tau phosphorylation. They enhance cholinergic neurotransmission by increasing acetylcholine levels and supporting cholinergic signaling through ChAT, VAChT, and cholinergic receptors (mAChR and nAChR). Furthermore, phytochemicals promote cellular homeostasis by preserving mitochondrial function and protein folding capacity (UPR), while mitigating oxidative stress (ROS). Abbreviations: Aβ—Amyloid-β; Acetyl-CoA—Acetyl coenzyme; ACh—Acetylcholine; AChE—Acetylcholinesterase; AD—Alzheimer’s disease; APP—Amyloid precursor protein; α-Syn—α-Synuclein; ChAT—Choline acetyltransferase; CDK-5—Cyclin-dependent kinase 5; EGCG—Epigallocatechin gallate; GSK—Glycogen synthase kinase 3; mAChR—Muscarinic acetylcholine receptor; nAChR—Nicotinic acetylcholine receptor; UPR—Unfolded protein response; VAChT—Vesicular acetylcholine transporter. This figure was created using Servier Medical Art (available at https://smart.servier.com/, accessed on 1 May 2025).

A noteworthy observation from the literature is that neurodegenerative diseases cover a wide range of conditions involving progressive deterioration and dysfunction of the nervous system, as well as mental and cognitive disorders such as depression, anxiety, and attention deficit disorders. Neurodegenerative diseases such as Alzheimer’s and Parkinson’s are increasingly recognized as multifactorial conditions. As shown in Figure 4, the neuroprotective mechanisms of phytochemicals in neurodegenerative diseases are highlighted, including pathways such as reducing oxidative stress, anti-inflammatory effects, and inhibiting protein aggregation. These pathways are affected by factors such as oxidative stress, chronic inflammation, neurotransmitter imbalances, mitochondrial dysfunction, and impaired neuroplasticity, as highlighted by the authors [165,166]. Phytochemicals such as curcumin, quercetin, resveratrol, and EGCG have been shown to interact with key molecular cascades, including the NF-κB, PI3K/Akt, ERK/CREB, and Nrf2/ARE pathways. These interactions may enhance synaptic resilience, reduce neuronal damage, and lead to improved behavioral outcomes, demonstrating a consistent pattern across various contexts [79,167].

This section provides an evidence-based overview of the role of specific phytochemicals in the prevention and treatment of various neurocognitive conditions. Particular emphasis is placed on the mechanisms by which these compounds modulate neurotransmission, neuroinflammation, and neurotrophic signaling. The potential of these compounds as components of integrative approaches to mental health and cognitive maintenance is also highlighted.

### 5.1. Phytochemicals as Multi-Target Agents in the Integrative Management of Depression and Mood Disorders

Current treatments for depression include various synthetic drugs with limitations such as side effects, targeting a single pathway, and slow onset of action [168]. A substantial body of research has demonstrated that many phytochemicals exert antidepressant-like effects by targeting key molecular pathways involved in neuroplasticity, neurotransmission, and immune regulation [169,170]. Several authors have argued that compounds such as curcumin, hesperidin, and quercetin activate the ERK-CREB-BDNF pathway, thereby increasing BDNF expression. BDNF plays a critical role in synaptic health, emotional regulation, and resilience to stress-related disorders [171,172].

Reduced levels of BDNF are often observed in patients with major depressive disorder (MDD), suggesting a central role in the pathophysiology of depression [173]. By promoting BDNF expression, these phytochemicals support neuronal survival, dendritic spine formation, and synaptic connectivity—fundamental processes that are often disrupted in depressive pathology [79]. Additionally, the inhibition of the MAO enzyme by phytochemicals such as hesperetin, quercetin, and kaempferol increases the synaptic availability of monoamines, including serotonin, dopamine, and norepinephrine [92]. This mechanism mirrors the pharmacological action of MAO inhibitors, a class of conventional antidepressants, and highlights the potential of phytochemicals to modulate monoaminergic transmission with a lower risk of the adverse effects commonly associated with synthetic agents.

In addition to their neurochemical effects, many phytochemicals possess anti-inflammatory properties, which are increasingly recognized as an integral part of depression treatment [168,174]. A common mechanism of action for compounds such as curcumin, apigenin, and resveratrol is the suppression of NF-κB, a key transcription factor that regulates pro-inflammatory cytokines such as IL-6, TNF-α, and IL-1β [175], which highlights the complexity of this phenomenon. Chronic low-grade neuroinflammation has been implicated in the pathogenesis of MDD, contributing to disruptions in neurotransmitter metabolism, neuroendocrine regulation, and neuronal plasticity [176]. By downregulating NF-κB and associated inflammatory pathways, phytochemicals contribute to the restoration of neuroimmune homeostasis and the alleviation of depressive symptoms, which aligns with trends observed in recent studies [168].

Emerging evidence also highlights the role of the gut–brain axis in mood disorders, suggesting that phytochemicals may exert antidepressant effects by modulating gut microbiota composition and reducing intestinal inflammation [177]. For instance, polyphenols such as EGCG and curcumin have been demonstrated to enhance the presence of beneficial microbiota (e.g., *Lactobacillus* and *Bifidobacterium*) while suppressing endotoxemia. These changes contribute to a reduction in both peripheral and central inflammation, supporting improved mood regulation [178,179]. The comparative results of the use of neuroactive phytochemicals in the treatment of depression and mood disorders are shown in Table 3.

Thus, the antidepressant-like properties of certain phytochemicals stem from their multi-targeted actions, which include modulation of neurotrophic factors, monoaminergic systems, inflammatory pathways, and gut–brain interactions. This positions them as promising candidates for the integrative treatment of depression and mood disorders.

### 5.2. Phytochemicals with Stress- and Anxiety-Modulating Properties

As discussed in previous research, there is substantial evidence to suggest that the anxiolytic effects of phytochemicals are often mediated by modulation of GABAergic transmission and regulation of the hypothalamic–pituitary–adrenal (HPA) axis [189]. Apigenin, a naturally occurring flavonoid found in *Matricaria chamomilla* (chamomile), has been shown to selectively bind to the benzodiazepine site of the GABA-A receptor. This enhances inhibitory neurotransmission and promotes a state of calm, without the sedative and addictive properties associated with synthetic anxiolytics, such as benzodiazepines [190,191].

Recent evidence has shown that chronic stress, characterized by sustained activation of the HPA axis, results in elevated cortisol levels and increased production of pro-inflammatory cytokines (IL-6 and TNF-α), as well as disrupting neuronal homeostasis in brain regions such as the hippocampus and prefrontal cortex [192]. Several phytochemicals have been shown to reduce stress by downregulating the NF-κB pathway, which is a key mediator of stress-induced neuroinflammation. Rosmarinic acid, for example, has been shown to suppress NF-κB activation, thereby reducing the expression of pro-inflammatory mediators, as well as modulating corticotropin-releasing hormone (CRH) and adrenocorticotropic hormone (ACTH) signaling. This leads to the homeostatic regulation of cortisol secretion [193,194]. Similarly, EGCG has been reported to attenuate stress-induced behavioral and biochemical changes by inhibiting glucocorticoid receptor overactivation and restoring neuronal antioxidant capacity through activation of the Nrf2/ARE pathway [195]. Furthermore, EGCG promotes hippocampal neurogenesis and preserves synaptic integrity in situations of chronic stress [196]. Table 4 shows the neuroactive phytochemicals involved in treating anxiety and chronic stress.

Importantly, phytochemicals may offer benefits comparable to those of standard pharmacotherapies, such as benzodiazepines or selective serotonin reuptake inhibitors (SSRIs), in terms of alleviating anxiety in preclinical and some clinical settings, while exhibiting fewer sedative, addictive, or metabolic side effects. However, direct head-to-head clinical trials are limited, and the magnitude of their effect relative to conventional drugs has yet to be systematically evaluated. This highlights the need for more comparative studies to establish their clinical significance.

Together, these phytochemicals provide a multifaceted approach to treating anxiety and chronic stress, as they modulate neurotransmitter systems, exert anti-inflammatory properties, and regulate endocrine responses. Their botanical origin, favorable safety profile, and ability to interact with multiple molecular targets underscore their therapeutic potential in treating stress-related neuropsychiatric disorders. Such disorders include generalized anxiety disorder, social anxiety disorder, and stress-related cognitive impairment. Conventional pharmacotherapy often falls short in terms of long-term safety and tolerability, as an earlier scientific team reported [92].

### 5.3. Phytochemicals in the Management of Cognitive Impairment and “Brain Fog”

Numerous studies have highlighted that cognitive deficits associated with chronic stress, systemic inflammation, or aging are strongly linked to synaptic dysfunction, oxidative damage, and neuroinflammation. These impair memory, attention, and executive function [198,199]. Flavonoids such as quercetin and EGCG have remarkable cognitive effects, mainly through activating the PI3K/Akt and Nrf2/ARE pathways [200,201]. Indeed, activation of these pathways promotes the expression of endogenous antioxidant enzymes, such as SOD, GPx, and catalase, thereby alleviating neuronal oxidative stress and supporting cellular resilience [202].

**Figure 5:**
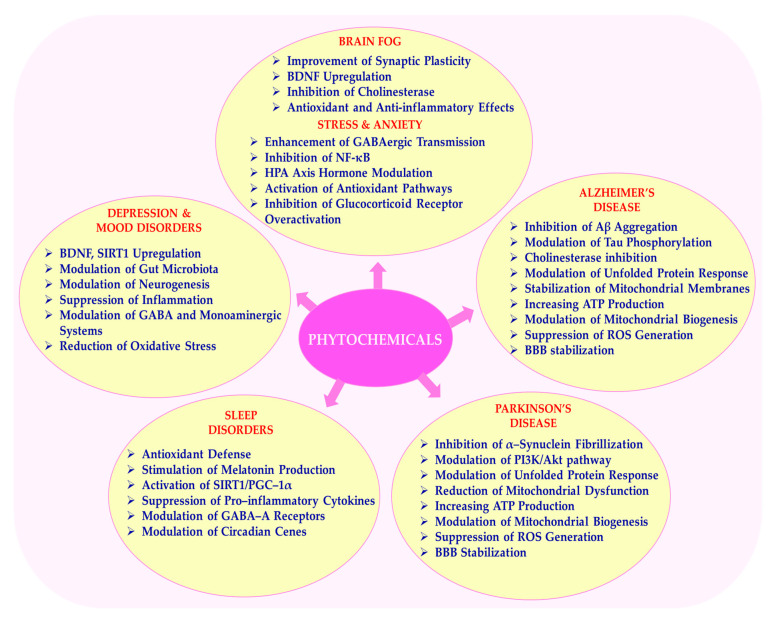
The neuroprotective and psychoprotective effects of phytochemicals in neurodegenerative and psychosomatic disorders. Phytochemicals promote neuronal survival and plasticity by upregulating BDNF and supporting PI3K/Akt signaling. They also maintain mitochondrial function via the PGC-1α and SIRT1 pathways. They modulate neurotransmission by regulating GABAergic signaling via GABA-A receptors, thereby alleviating anxiety and stress-related responses. Furthermore, phytochemicals protect the blood–brain barrier, reduce reactive oxygen species (ROS) production, inhibit proinflammatory NF-κB signaling, and regulate the hypothalamic–pituitary–adrenal (HPA) axis, thereby mitigating the effects of chronic stress. Abbreviations: Aβ—Amyloid-β; Akt—Protein kinase B (PKB); ATP—Adenosine triphosphate; BBB—Blood–brain barrier; BDNF—Brain-derived neurotrophic factor; GABA-A receptor—γ-Aminobutyric acid sub-type A receptor; GABA—γ-aminobutyric acid; HPA axis—Hypothalamic–pituitary–adrenal axis; NF-κB—Nuclear Factor-kappa B; PGC-1α—Peroxisome proliferator-activated receptor gamma coactivator 1-alpha; PI3K—Phosphatidylinositol 3-kinase; ROS—Reactive oxygen species; SIRT1—Silent information regulator 1.

In line with the findings of Li et al. (2025) and Hajialyani et al. (2019), curcumin and hesperidin have demonstrated the ability to restore synaptic plasticity by increasing BDNF expression via activation of the ERK–CREB pathway. This improves both hippocampal function and long-term potentiation, which are key processes underlying learning and memory [203,204]. Furthermore, both compounds significantly reduce ROS accumulation and suppress the production of pro-inflammatory cytokines such as IL-6 and TNF-α, which contribute to neuroinflammation and cognitive impairment commonly referred to as ‘brain fog’ [203,204,205]. Figure 5 illustrates phytochemicals in neurodegenerative and psychosomatic disorders.

Emerging evidence also suggests that rosmarinic acid may enhance cholinergic transmission by inhibiting acetylcholinesterase activity, thereby improving attention and short-term memory in cases of stress-related cognitive dysfunction [206]. Overall, these neuroactive phytochemicals demonstrate significant therapeutic potential in alleviating cognitive impairments linked to modern lifestyle stressors and age-related neurodegenerative processes (Table 5).

### 5.4. Phytochemical Approaches to Alleviate Neurodegenerative Diseases

Numerous studies have highlighted the importance of neurodegenerative diseases such as AD and PD, which are characterized by progressive neuronal loss, protein misfolding, mitochondrial dysfunction, and oxidative stress. These diseases ultimately lead to cognitive and motor impairments, as demonstrated in studies [215,216]. Therefore, recent research has identified phytochemicals as promising neuroprotective agents due to their ability to modulate multiple molecular and cellular pathways involved in the pathogenesis of these disorders, including neuroinflammation, oxidative damage, and impaired proteostasis [81].

One of the pathological hallmarks of Alzheimer’s disease is the accumulation of Aβ peptides, which aggregate into extracellular plaques and trigger a cascade of neurotoxic events, including oxidative stress, mitochondrial dysfunction, and synaptic loss. Additionally, hyperphosphorylation of the tau protein disrupts microtubule stability and axonal transport, leading to the formation of intracellular neurofibrillary tangles [217]. Several phytochemicals, including curcumin, resveratrol, and EGCG, have been shown to inhibit Aβ aggregation and modulate tau phosphorylation by regulating the activity of key enzymes, such as glycogen synthase kinase-3β (GSK-3β), and protein phosphatases involved in tau metabolism, as demonstrated in studies [218,219]. These compounds exert their neuroprotective effects through multiple validated pathways. These include activating Nrf2/ARE signaling to enhance antioxidant responses, inhibiting NF-κB to suppress neuroinflammation, and modulating CREB-mediated transcription to support neuronal survival and synaptic plasticity. Furthermore, compounds such as huperzine A enhance cholinergic neurotransmission by inhibiting acetylcholinesterase, thereby improving memory and learning performance in preclinical AD models [220,221].

A noteworthy observation from the literature is that PD is characterized primarily by the degeneration of dopaminergic neurons in the *substantia nigra* and the accumulation of α-synuclein aggregates in the form of Lewy bodies [222]. Phytochemicals such as baicalein, naringenin, and salidroside have been reported to reduce α-synuclein toxicity and enhance dopaminergic neuroprotection, primarily by activating the Nrf2/ARE antioxidant pathway, suppressing NF-κB-driven inflammatory signaling, and stabilizing mitochondrial function [223,224,225]. In addition, these compounds may modulate the MAO-B enzyme, thereby reducing oxidative damage caused by dopamine metabolism [226,227].

Both AD and PD are associated with impaired protein homeostasis and mitochondrial dysfunction [216]. Polyphenolic compounds such as quercetin, EGCG, and luteolin help maintain protein folding capacity by modulating the unfolded protein response and increasing proteasomal activity. They also stabilize mitochondrial membranes, increase ATP production, and attenuate ROS generation, thereby preserving neuronal integrity, as shown in studies [228,229]. Overall, across multiple neurodegenerative models, Nrf2/ARE activation appears to be the most consistently validated pathway for antioxidant and cytoprotective effects. Meanwhile, NF-κB inhibition primarily mediates anti-inflammatory actions, and CREB modulation supports synaptic plasticity and memory functions. These pathways collectively underlie the multi-targeted neuroprotective mechanisms of phytochemicals. The protective roles of selected phytochemicals in combating neurodegenerative diseases are presented in Table 6.

Taken together, these findings suggest that phytochemicals offer a multi-targeted approach to mitigating the neuropathological features of neurodegenerative diseases, representing a valuable addition to existing therapeutic strategies [237].

### 5.5. Plant-Derived Agents in the Management of Sleep Disorders

Sleep disorders, including insomnia and circadian rhythm disorders, are a growing global health concern. These disorders are often associated with anxiety, depression, and neurodegenerative diseases [238]. Recent evidence suggests that various phytochemicals may modulate sleep–wake cycles and restore circadian homeostasis by regulating melatonin, influencing GABAergic neurotransmission, and exhibiting anti-inflammatory activity (Table 7).

One of the key mechanisms of phytochemical action is the modulation of melatonin synthesis, which is crucial for regulating circadian rhythms [251]. Myricetin, a major constituent of the cortex of *Myrica rubra* (Lour.) Siebold & Zucc., inhibits the activity of serotonin N-acetyltransferase (AANAT; acetyl-CoA: arylalkylamine N-acetyltransferase, EC 2.3.1.87), reduces nocturnal melatonin levels, and alters locomotor activity in rats. This suggests that it has the potential to enhance nocturnal alertness by modulating circadian rhythms [252]. Additionally, phytochemicals such as resveratrol can influence the suprachiasmatic nucleus (SCN), the central circadian pacemaker, via the SIRT1/PGC-1α pathway, thereby improving sleep quality and circadian alignment [243]. Several anxiolytic and sedative phytochemicals act by modulating GABA-A receptors. Apigenin, a bioactive flavonoid found in chamomile, binds to the benzodiazepine site of the GABA-A receptor, producing a sedative effect without the adverse side effects commonly associated with synthetic hypnotics [190,253]. Similarly, valerenic acid derivatives found in *Valeriana officinalis* (valerian root) enhance GABAergic transmission through allosteric modulation, thereby reducing sleep latency and prolonging sleep duration [254].

Phytochemicals may promote better sleep by reducing inflammation, which interferes with normal sleep patterns. Poor sleep quality has been linked to chronic low-grade neuroinflammation, as demonstrated by Manchanda et al. [255]. Compounds such as EGCG and rosmarinic acid have been shown to suppress pro-inflammatory cytokines, such as TNF-α and IL-6, by inhibiting the NF-κB pathway [163,256]. Taken together, phytochemicals provide a multi-targeted approach to sleep regulation by modulating hormonal, neurochemical, and inflammatory pathways. These natural products show promise as an alternative or additional option to conventional hypnotics, particularly for individuals with comorbid psychiatric or neurodegenerative disorders.

### 5.6. Plant-Based Neurotherapeutics for Attention Deficit and Executive Dysfunction

Attentional and executive dysfunction, which is commonly observed in conditions such as attention-deficit/hyperactivity disorder (ADHD), is characterized by impairments in sustained attention, working memory, and inhibitory control [257]. These cognitive deficits are closely linked to dysregulations in catecholaminergic neurotransmission, particularly involving the dopamine and norepinephrine pathways in the prefrontal cortex (PFC) [258].

Several phytochemicals have shown potential in modulating catecholaminergic neurotransmission (see Table 8). For example, *Rhodiola rosea*, a plant with adaptogenic properties, contains salidroside and rosavin, which have been reported to enhance dopaminergic transmission and support PFC-dependent cognitive functions [259]. Similarly, ginsenosides derived from ginseng have been shown to modulate dopaminergic and noradrenergic signaling by inhibiting catecholamine reuptake and increasing receptor sensitivity [260].

In addition, polyphenols such as quercetin and EGCG exhibit anti-inflammatory and antioxidant properties that are relevant to ADHD-like phenotypes. This is because low-grade neuroinflammation and oxidative stress are now widely accepted as contributing factors in executive dysfunction [267,268]. These compounds reduce microglial activation and suppress pro-inflammatory cytokines, such as IL-6 and TNF-α. They also activate the Nrf2-ARE pathway, thereby enhancing cellular resilience and exerting neuroprotective effects [269].

Another promising class of phytochemicals is those with cognitive-enhancing (nootropic) properties, such as bacosides from *Bacopa monnieri* and withanolides from *Withania somnifera*. These compounds have been shown to improve attention span, working memory, and processing speed by increasing BDNF expression, promoting synaptogenesis, and modulating cholinergic signaling [264,265,266]. Taken together, these findings suggest that selected phytochemicals, particularly polyphenols and adaptogens, may complement conventional therapies for attention deficit and executive dysfunction by targeting neurotransmitter modulation, neuroinflammation, and cognitive resilience.

In conclusion, the growing body of evidence highlights the potential of phytochemicals, particularly polyphenols, adaptogens, and nootropic compounds, in treating attention deficit and executive dysfunction. These natural products offer a multifaceted approach to improving cognitive function by modulating neurotransmitter systems, reducing neuroinflammation, and enhancing cognitive resilience. Their ability to target key physiological pathways, such as dopaminergic and noradrenergic signaling, as well as inflammatory processes, makes them a promising addition to conventional treatments, particularly for individuals with disorders such as ADHD and associated cognitive impairments. Further investigation of their efficacy and safety in clinical settings is required to fully understand their potential in cognitive health.

### 5.7. Phytochemicals in Cognitive Aging and Dementia Prevention

The data suggest a significant correlation between age-related cognitive decline and the multifactorial development of dementia involving oxidative stress, chronic neuroinflammation, synaptic dysfunction, and epigenetic dysregulation [270]. Therefore, phytochemicals have emerged as a promising way to mitigate these processes due to their neuroprotective, antioxidant, and anti-inflammatory properties (Table 9).

Long-term supplementation with polyphenols and other phytochemicals, such as resveratrol, EGCG, and curcumin, has been associated with improved cognitive outcomes and delayed neurodegeneration in aging populations, providing a basis for future theoretical and empirical work. Specifically, these compounds modulate key signaling pathways, such as PI3K/Akt, Nrf2/ARE, and SIRT1, thereby enhancing neuronal survival, upregulating antioxidant defenses, and reducing cellular senescence [271,272,273,274].

The anti-aging potential of phytochemicals is also attributed to their ability to modulate epigenetic mechanisms. For instance, resveratrol activates SIRT1, a NAD^+^-dependent deacetylase that plays a role in longevity and neuronal resilience [281]. Additionally, curcumin and sulforaphane have been shown to affect DNA methylation and histone acetylation, thereby altering the transcription of genes involved in cognition and brain plasticity [282,283]. Importantly, phytochemicals can inhibit the production of pro-inflammatory cytokines (e.g., IL-1β, IL-6, and TNF-α) and reduce microglial activation. Both of these are key contributors to the neurodegenerative environment in the aging brain [131]. By mitigating oxidative damage and preserving synaptic integrity, phytochemicals represent a promising strategy for preventing dementia and age-related cognitive impairment [284,285].

There is a growing body of evidence that supports the role of phytochemicals in mitigating age-related cognitive decline and the progression of dementia. Through a combination of antioxidant, anti-inflammatory, and epigenetic mechanisms, compounds such as resveratrol, curcumin, EGCG, and sulforaphane target the underlying pathophysiology of brain aging. Their ability to preserve synaptic function, reduce neuroinflammation, and promote neuronal resilience highlights their potential as preventive or adjunctive agents in the treatment of cognitive aging and neurodegenerative disorders. Further research is needed to determine their therapeutic efficacy, bioavailability, and long-term safety in aging populations.

## 6. Targeting Prodromal Stages of Neurodegenerative Diseases with Plant-Based Compounds

Neurodegenerative diseases, including Alzheimer’s and Parkinson’s, are characterized by a multifactorial etiology involving abnormal protein aggregation, mitochondrial dysfunction, oxidative stress, excitotoxicity, and chronic neuroinflammation [286]. Notably, pathological changes often begin decades before the onset of clinical symptoms, emphasizing the urgent need for preventive strategies and early therapeutic intervention [69]. Phytochemicals have demonstrated promising neuroprotective effects by targeting the early stages of neurodegenerative processes. For example, EGCG has been shown to prevent the aggregation of amyloid-β and α-synuclein by stabilizing their native conformations and enhancing proteasomal and autophagic clearance mechanisms [287] (Table 10). Furthermore, EGCG suppresses Aβ-induced neurotoxicity by inhibiting ROS generation and lipid peroxidation [149].

Curcumin, a polyphenol derived from turmeric, modulates apoptotic signaling by regulating the Bax/Bcl-2 ratio. This inhibits cytochrome c release and prevents mitochondrial membrane depolarization [295]. Curcumin also exhibits potent anti-inflammatory activity by downregulating NF-κB and pro-inflammatory cytokines (e.g., TNF-α and IL-1β), contributing to neuronal resilience in the early stages of neurodegeneration [114]. Quercetin, a flavonoid found in several fruits and vegetables, activates SIRT1, a NAD^+^-dependent deacetylase associated with longevity and neuronal survival, adding depth to our understanding of the subject [125]. Activation of SIRT1 supports mitochondrial biogenesis, DNA repair, and chromatin remodeling, while also regulating key transcription factors, such as PGC-1α and FOXO3a, that mediate cellular stress resistance [296].

Thus, these mechanisms demonstrate that phytochemicals provide a multi-targeted approach to slowing the early progression of neurodegenerative diseases. By targeting key molecular events at the prodromal stage, phytochemicals have the potential to manage symptoms and modify disease trajectories, preserving cognitive function [297].

## 7. Gut–Brain Axis as a Mediator of Phytochemical Activity

Emerging evidence suggests that the gut–brain axis (GBA) plays a pivotal role in mediating the neurological effects of phytochemicals [177]. This bidirectional communication network between the central and enteric nervous systems is significantly modulated by immune, neural, endocrine, and metabolic pathways [298]. Disruption of the GBA has been linked to various neuropsychiatric and neurodegenerative disorders, such as depression, anxiety, and Alzheimer’s disease [299].

Polyphenols such as rosmarinic acid, quercetin, and curcumin have been shown to modulate the composition and function of the gut microbiota, promoting the growth of beneficial bacteria such as *Lactobacillus* and *Bifidobacterium* [300]. These shifts in the microbiome, induced by phytochemicals, are accompanied by specific functional changes. These include enhanced production of short-chain fatty acids (SCFAs), such as butyrate, propionate, and acetate. These SCFAs serve as key modulators of intestinal and systemic immunity. They also maintain gut barrier integrity and influence microglial activation in the brain [301]. Furthermore, polyphenol-stimulated beneficial microbes facilitate the synthesis of neurotransmitter precursors, including GABA, serotonin, dopamine, and tryptophan metabolites. These directly impact mood regulation, cognitive function, and synaptic plasticity [302].

It has also been observed that phytochemicals improve intestinal epithelial integrity by upregulating tight junction proteins such as occludin and zonula occludens-1. This reduces intestinal permeability and prevents the systemic translocation of lipopolysaccharide (LPS) [303]. This subsequently reduces systemic endotoxemia, which is associated with reduced activation of microglial cells and suppression of pro-inflammatory pathways, such as the TLR4/NF-κB signaling cascade, in the brain [304]. These immunomodulatory effects help attenuate neuroinflammation and are associated with improved behavioral outcomes in the context of stress and mood disorders [131]. Consequently, the effects of phytochemicals extend beyond the central nervous system, highlighting the importance of gastrointestinal health in maintaining overall neurological well-being (Table 11). Phytochemicals can exert more targeted effects on the gut–brain axis by specifically modulating SCFA production and neurotransmitter precursor availability. This represents a mechanistic link between dietary interventions, microbiome composition, and neurocognitive health. Therefore, targeting the gut microbiota may be a promising approach for preventing and treating cognitive decline and psychiatric disorders.

## 8. Microbiota–Gut–Brain Communication as a Pathway for Phytochemical Action

Numerous studies have demonstrated the importance of microbiome-mediated biotransformation of phytochemicals [311,312]. Indeed, the gut microbiota plays a crucial role in converting dietary polyphenols, flavonoids, and alkaloids into bioactive metabolites that are more easily absorbed and have greater systemic bioavailability and therapeutic efficacy. This enzymatic conversion often results in smaller, more lipophilic compounds that can cross both the intestinal and blood–brain barriers and exert effects on the central nervous system [313]. A comprehensive understanding of the gut–brain–phytochemical triad could greatly advance precision medicine and the development of personalized nutritional strategies for mental healthcare (Table 12).

Individuals with gut dysbiosis, which is characterized by reduced microbial diversity or the overgrowth of pathogenic species, may not benefit fully from specific herbal therapies due to impaired microbial enzymatic activity [321]. Consequently, co-administering probiotics, prebiotics, or synbiotics alongside phytochemicals has emerged as an approach to optimize therapeutic outcomes in conditions such as major depressive disorder, generalized anxiety disorder, and age-related cognitive decline [322]. For instance, combining *Lactobacillus rhamnosus* with a polyphenol-rich diet has been shown to increase anxiolytic effects by modulating the GABA receptor [323].

As research progresses, elucidating the complex interactions between diet, the microbiome, and phytochemicals may become a cornerstone of future neuropsychiatric therapeutics. This highlights the importance of integrating nutritional science, microbiota modulation, and phytotherapeutic compounds into comprehensive mental health strategies. Many polyphenols, including quercetin, curcumin, EGCG, and hesperidin, are poorly absorbed in their native forms [324]. However, microbial enzymes, particularly those found in the colon, can degrade these compounds into smaller, more bioactive derivatives. For instance, *Bacteroides* and *Clostridium* species can metabolize hesperidin to hesperetin, which readily crosses the BBB and exhibits neuroprotective and anti-inflammatory properties, as reported by Khan et al. [325]. Thus, the gut microbiota acts as a metabolic gatekeeper, shaping the pharmacokinetics, potency, and neurobiological effects of phytochemicals [326].

In addition to biotransformation, gut microbes synthesize neuroactive compounds that profoundly affect brain function. SCFAs, such as butyrate, acetate, and propionate, are produced through the fermentation of dietary fiber and polyphenols. Numerous studies have highlighted the importance of these SCFAs, which have been shown to improve blood–brain barrier integrity, regulate microglial homeostasis, and stimulate BDNF expression [327]. Furthermore, SCFAs influence the release of serotonin in the gastrointestinal tract and indirectly modulate central serotonergic tone, thereby affecting mood and behavior [328]. Furthermore, the microbial metabolism of ellagitannins and flavonoids yields phenolic acids and urolithins—compounds that reduce oxidative stress, alleviate neuroinflammation, and support mitochondrial health [329].

The bidirectional nature of the gut–brain axis considerably complicates the interplay between the microbiota and the central nervous system. This communication network includes neural (e.g., the vagus nerve), endocrine (e.g., cortisol and ghrelin), immune (cytokines), and metabolic (e.g., SCFAs and neurotransmitters) pathways [298,330]. Several landmark studies have shaped our current understanding that microbial signals originating in the gut can influence brain function by modulating neurotransmission, synaptic plasticity, and behavior [331]. Conversely, psychological stress can disrupt gastrointestinal function, promote mucosal inflammation, and destabilize microbial communities [332]. Chronic stress has been shown to be associated with increased intestinal permeability (‘leaky gut’), facilitating the translocation of lipopolysaccharide into the systemic circulation. This, in turn, exacerbates neuroinflammation and contributes to the progression of depressive symptoms [333]. Phytochemicals such as rosmarinic acid and baicalin have been shown to stabilize tight junction proteins, attenuate LPS-induced inflammation, and maintain gut–brain homeostasis [334,335].

The role of phytochemicals as prebiotics and microbial modulators has received increasing attention in the fields of neuropharmacology and nutritional science. Therefore, several neuroactive phytochemicals not only exert direct effects on the CNS but also selectively promote the proliferation of beneficial bacteria [336]. For instance, EGCG and quercetin, which are found in apples, onions, and berries, have been demonstrated to enhance the presence of *Lactobacillus* and *Bifidobacterium*, which are linked to enhanced gut and mental health [317]. This beneficial shift in the gut microbial community increases the production of SCFAs, tryptophan, and other neurotransmitter precursors, thereby supporting the biosynthesis of serotonin, the regulation of mood, and cognitive resilience [337]. In summary, these findings emphasize the pivotal role of the gut microbiota in shaping the pharmacodynamics of phytochemicals. They also suggest that future strategies to promote brain health may benefit from considering the composition of the individual microbiome.

## 9. Current Challenges and Future Perspectives in Neurophytochemistry

The clinical application of phytochemicals presents several significant challenges, primarily concerning their bioavailability, optimal dosage, and pharmacokinetics. Their poor systemic bioavailability is a major limitation to their therapeutic efficacy, largely due to low aqueous solubility, rapid metabolic degradation, and limited gastrointestinal absorption [338]. Despite demonstrating considerable in vitro potency, compounds such as curcumin and quercetin undergo extensive first-pass hepatic metabolism, which significantly reduces their bioactivity in vivo [339]. To overcome these barriers, innovative strategies are being explored, including the use of nanoparticle-based drug delivery systems, liposomal encapsulation, phytosome formulations, and the co-administration of bioenhancers. For instance, piperine has been demonstrated to substantially enhance the bioavailability of curcumin by inhibiting glucuronidation [340,341]. Furthermore, the dose–response relationship is a critical consideration for clinical translation, as many phytochemicals have narrow therapeutic windows that can vary depending on individual metabolic profiles, the composition of the gut microbiota, and genetic polymorphisms that affect drug metabolism [342]. Understanding these factors, along with formulation strategies, is essential to optimizing in vivo efficacy and BDNF-modulating potential.

Another important yet complex issue is the potential for phytochemical–drug interactions and synergistic effects, given that many phytochemicals can enhance the efficacy of both natural products and conventional drugs. For instance, EGCG has been demonstrated to amplify the neuroprotective properties of L-DOPA in Parkinson’s disease models by mitigating oxidative stress and bolstering dopaminergic function [149,150].

Beyond interactions with drugs, there is growing evidence that combinations of phytochemicals, as found in polyphenol-rich diets, can have a greater effect than individual compounds. For instance, a combination of resveratrol and quercetin has been shown to reduce amyloid-β aggregation more effectively and improve mitochondrial function in Alzheimer’s disease models than either compound alone [343]. Similarly, co-administering curcumin and piperine increases curcumin bioavailability and enhances its anti-inflammatory and antioxidant effects in vivo [344]. Flavonoid–stilbene (e.g., quercetin plus resveratrol) and flavonoid–catechin (e.g., EGCG plus luteolin) mixtures have demonstrated additive or synergistic effects on Nrf2/ARE activation, NF-κB inhibition, and CREB-mediated synaptic plasticity. This leads to improved learning and memory outcomes in rodent models of neurodegeneration [343,344,345]. These results imply that dietary patterns rich in various polyphenols could provide broader, multi-target neuroprotection than supplementation with individual phytochemicals, while also enabling the use of lower effective doses [346].

However, such synergy may pose potential safety risks. For example, flavonoids such as quercetin can inhibit cytochrome P450 enzymes, particularly CYP3A4 and CYP2C9, which could affect the metabolism of several prescription drugs, including antidepressants, anticoagulants, and anticonvulsants [347]. These interactions emphasize the importance of thorough evaluation, particularly in cases of polypharmacy and among vulnerable groups such as the elderly or individuals with comorbidities [348].

Another major barrier to integrating phytochemicals into clinical practice is the lack of standardized, high-quality clinical trials [349]. Although numerous preclinical studies have reported promising neuroprotective and psychotropic effects, the translation of these findings into clinical benefits remains limited. Variability in the composition of botanical extracts, non-standardized dosing regimens, and inconsistent outcome measures hinder data comparability and reproducibility [350]. Furthermore, small sample sizes, brief intervention durations, and underreporting of adverse effects further undermine the reliability of clinical evidence [351]. To advance the field, there is an urgent need for harmonized methodologies, standardized extract preparations, pharmacokinetic modeling, and long-term safety assessments.

Integrating phytochemicals into functional foods, dietary supplements, and personalized nutrition represents a promising frontier in neurophytotherapy; hence, the development of phytochemical-enriched functional products, such as polyphenol-infused beverages, nootropic capsules, and fortified nutritional bars, is gaining momentum as a strategy to improve both adherence and bioefficacy [352,353]. In parallel, advances in personalized nutrition based on gut microbiome sequencing, metabolomics, and nutrigenomics may enable phytochemical interventions to be tailored to individual physiological profiles [354]. This precision-based approach could improve therapeutic outcomes by taking into account variations in absorption, metabolism, and microbiota–phytochemical interactions.

Compared to conventional pharmacological agents, phytochemicals often exhibit a favorable safety profile, with lower toxicity and fewer side effects, making them appealing as complementary or stand-alone therapeutic options [7]. Nevertheless, safety aspects must be carefully considered. There have been reports of hepatotoxicity related to high-dose green tea extracts (EGCG), gastrointestinal irritation associated with curcumin supplementation, and photosensitivity or cytochrome P450-mediated drug interactions involving *Hypericum perforatum* (St John’s wort). These cases highlight the importance of monitoring adverse events [355,356,357]. Furthermore, phytochemicals may interfere with commonly prescribed drugs (e.g., anticoagulants, antidepressants, and antihypertensives), raising concerns about drug–nutrient interactions [358]. While most reports indicate good tolerability, the relatively short duration and small sample sizes of these studies limit the detection of long-term or rare adverse effects. These issues underscore the need for rigorous safety evaluation, pharmacovigilance, and the development of evidence-based guidelines before phytochemicals can be more widely used in clinical practice.

In summary, integrating phytochemicals into mainstream neurotherapeutic strategies shows great promise, provided their use is guided by rigorous scientific evidence, comprehensive safety assessments, and individualized clinical application. Although these compounds are naturally occurring, their pharmacodynamic and pharmacokinetic properties vary widely, and therapeutic efficacy depends on the form, context, and duration of use. Future advances in neurophytochemistry depend on interdisciplinary collaboration between phytochemists, neuroscientists, clinical pharmacologists, and nutritionists. The ultimate goal is to position phytochemicals as evidence-based adjuncts to conventional therapies and as preventive agents for long-term cognitive and mental well-being.

## 10. Conclusions

Recent advances in phytotherapy have highlighted the therapeutic potential of plant-derived compounds in supporting brain health, particularly in the treatment of mood disorders, cognitive decline, anxiety, and neurodegenerative conditions. Well-characterized molecular pathways through which phytochemicals such as apigenin, curcumin, EGCG, quercetin, hesperidin, and rosmarinic acid exert their effects include modulation of BDNF expression, inhibition of NF-κB-mediated inflammation, enhancement of antioxidant defenses via activation of the Nrf2 pathway, and regulation of neurotransmitter systems such as GABA, serotonin, and dopamine.

It is important to recognize the significant influence of the gut microbiota on the metabolism, bioactivity, and, ultimately, the efficacy of these compounds. The bidirectional interaction between phytochemicals and the microbiome involves the modulation of microbial composition by specific phytochemicals and the conversion of phytochemicals into bioactive metabolites by microbial enzymes. This gut–brain–phytochemical axis can enhance or impair therapeutic outcomes, underlining the need for an integrated systems approach to neurophytotherapy research.

However, there are still several challenges to overcome, including the low bioavailability of many phytochemicals, variability in pharmacokinetics, differences in gut microbiota profiles between individuals, and potential interactions with conventional pharmaceuticals. Additionally, the absence of standardized, large-scale clinical trials, consistent dosing guidelines, and long-term safety data continues to hinder the application of preclinical findings in clinical practice.

Despite these limitations, phytochemicals show great promise as adjuvants in the development of functional foods, nutraceuticals, and personalized nutritional strategies to promote mental and neurological health. To exploit their full neuroprotective potential, future efforts should focus on optimizing delivery systems (e.g., nanoformulations and encapsulation techniques), integrating phytotherapy with microbiome-targeted interventions, and validating efficacy and safety through rigorous, high-quality clinical research. Consequently, phytochemicals could become an integral component of holistic, evidence-based strategies for promoting mental well-being and preventing and treating neurological disorders.

## Figures and Tables

**Table 1:** Phytochemicals with neuroactive properties.

Phytochemical Groups	Representative Compounds	Mechanisms of Action	Dietary Sources	References
Flavonoids	Quercetin, Luteolin, Kaempferol, Prunin, Isorhamnetin	Antioxidant activity, inhibition of neuroinflammation, modulation of ERK/PI3K/Akt/NF-κB signaling, enhancement of neuroplasticity	Apples, onions, parsley, celery, kale, broccoli, berries, green tea	[28,29,30,31,32,33,53,54,55]
Alkaloids	Caffeine, Huperzine A	Adenosine receptor antagonism, acetylcholinesterase inhibition, cognitive stimulation, neuroprotection	Coffee, tea, cocoa, *Huperzia serrata* extract	[34,56,57]
Terpenoids	Curcumin, Ginkgolides	Anti-inflammatory and antioxidant effects, modulation of amyloid-beta aggregation, improved cerebral blood flow	Turmeric, *Ginkgo biloba* leaves	[39,58,59,60,61]
Polyphenols	Resveratrol, EGCG	Activation of sirtuins, mitochondrial biogenesis, BDNF upregulation, modulation of GABAergic and dopaminergic systems	Red grapes, red wine, peanuts, green tea	[45,46,62,63]
Other sources	Anthocyanins, ellagic acid, ferulic acid	Free radical scavenging, support of synaptic integrity, gene expression modulation	Berries, pomegranates, whole grains, citrus fruits	[64,65,66,67]

**Table 2:** Major neuroactive phytochemicals, their molecular mechanisms, and their neurological relevance.

Phytochemicals	Dietary Sources	Main Molecular Mechanisms	Neuroprotective Functions	Associated Neurological Conditions	References
Apigenin	Chamomile, parsley, celery	Binds GABA-A receptor (benzodiazepine site); inhibits NF-κB; activates Nrf2/ARE	Anxiolytic; anti-inflammatory; antioxidant	Anxiety disorders, neuroinflammation, insomnia	[134,135,136,137,138,139]
Hesperidin	Citrus fruits (oranges, lemons)	Activates CREB-BDNF pathway; inhibits MAO; suppresses TLR4/NF-κB; stabilizes BBB	Enhances neurogenesis; antidepressant; anti-inflammatory	Depression, neurodegeneration, vascular dementia	[140,141,142,143,144]
EGCG	Green tea	Activates Nrf2; inhibits amyloid-β and α-synuclein aggregation; modulates PI3K/Akt and ERK1/2	Antioxidant; anti-amyloidogenic; neurotrophic	Alzheimer’s disease, Parkinson’s disease, oxidative stress	[145,146,147,148,149,150]
Curcumin	Turmeric	Activates Nrf2/ARE; inhibits NF-κB, COX-2, iNOS; enhances BDNF via ERK/CREB; modulates Bcl-2/Bax	Antioxidant; anti-inflammatory; anti-apoptotic	Alzheimer’s disease, depression, cognitive decline	[114,151,152,153,154,155]
Quercetin	Onions, apples, berries, kale	Activates SIRT1 and Nrf2; inhibits lipid peroxidation; suppresses TLR4 microglial activity	Antioxidant; anti-inflammatory; mitochondrial support	Cognitive impairment, neuroinflammation, aging brain	[156,157,158,159,160]
Rosmarinic acid	Rosemary, lemon balm, mint	Upregulates claudin-5 and occludin; modulates glutamate receptors; inhibits COX-2, TNF-α	BBB protection; anti-excitotoxic; anti-inflammatory	Epilepsy, neuroinflammation, stress-related disorders	[161,162,163,164]

**Table 3:** Neuroactive phytochemicals as therapeutic agents for depression and mood disorders.

Phytochemicals	Natural Sources	Mechanisms of Action	Neurobiological Targets	Relevant Pathways	Potential Benefits	References
Curcumin	Turmeric (*Curcuma longa*)	Enhances antioxidant defense, reduces neuroinflammation, modulates neurogenesis	BDNF, NF-κB, MAO	ERK–CREB–BDNF, Nrf2/ARE, NF-κB	Improves mood, reduces oxidative stress, supports synaptic plasticity	[26,180]
Hesperidin	Citrus fruits (oranges, lemons)	Increases BDNF, modulates monoamine levels, reduces inflammation	BDNF, MAO, NF-κB	CREB, TLR4/NF-κB	Antidepressant effect, promotes neuroplasticity	[181,182,183]
Quercetin	Apples, onions, berries	Inhibits MAO, reduces microglial activation, activates SIRT1	MAO, NF-κB, SIRT1	Nrf2, TLR4/NF-κB	Enhances mood, reduces neuroinflammation, supports mitochondrial function	[184,185]
Apigenin	Chamomile, parsley, celery	Suppresses inflammation, modulates GABA and monoaminergic systems	NF-κB, GABA receptors	NF-κB, benzodiazepine site modulation	Reduces anxiety and depressive symptoms	[183,186]
EGCG	Green tea	Modulates gut microbiota, reduces oxidative and inflammatory stress	Nrf2, microbiota, cytokines	JNK, NF-κB, Nrf2	Supports gut–brain axis, alleviates depression	[187,188]

**Table 4:** Neuroactive phytochemicals with stress- and anxiety-modulating properties.

Phytochem-icals	Plant Source	Mechanism of Action	Molecular Targets	Neuroprotective/Anxiolytic Effects	Relevance to Anxiety and Stress	References
Apigenin	Chamomile (*Matricaria chamomilla*)	Enhances GABAergic transmission via benzodiazepine site	GABA-A receptor	Anxiolytic, promotes relaxation	Reduces anxiety without sedative side effects	[191,197]
Rosmarinic acid	Rosemary (*Rosmarinus officinalis*), Lemon balm (*Melissa officinalis*)	Suppresses NF-κB, modulates HPA axis hormones	NF-κB, CRH, ACTH	Reduces inflammation, stabilizes cortisol	Mitigates neuroinflammation and HPA axis overactivation	[193,194]
EGCG (Epigallocatechin-3-gallate)	Green tea (*Camellia sinensis*)	Inhibits glucocorticoid receptor overactivation, activates antioxidant pathways	Nrf2/ARE, glucocorticoid receptor	Reduces oxidative stress, supports neurogenesis	Counters chronic stress and improves cognitive resilience	[195,196]

**Table 5:** Neuroprotective effects of selected phytochemicals on cognitive impairment and the “brain fog” condition.

Phytochemicals	Primary Mechanisms of Action	Targeted Pathways/Molecules	Cognitive Benefits	References
Quercetin	Antioxidant, anti-inflammatory, neuroprotective	PI3K/Akt, Nrf2/ARE, IL-6, TNF-α	Reduces oxidative stress and inflammation; enhances memory and attention	[207]
EGCG	Antioxidant, protein aggregation inhibition	Nrf2/ARE, PI3K/Akt, SOD, GPx	Improves neuronal survival; protects against cognitive decline	[208]
Curcumin	Enhances synaptic plasticity, antioxidant, anti-inflammatory	ERK/CREB/BDNF, IL-6, TNF-α	Improves learning and memory; reduces mental fatigue	[209,210]
Hesperidin	BDNF upregulation, anti-inflammatory, neurotrophic	ERK/CREB/BDNF, ROS, IL-6	Restores cognitive flexibility; supports memory retention	[211,212]
Rosmarinic acid	Cholinesterase inhibition, antioxidant	AChE, TNF-α	Improves attention and short-term memory; reduces cognitive fatigue	[213,214]

**Table 6:** Selected phytochemicals and their neuroprotective effects in the treatment of neurodegenerative diseases.

Phytochemicals	Primary Mechanism of Action	Targeted Pathology	Observed Effects	References
Curcumin	Inhibition of amyloid-β aggregation; modulation of tau phosphorylation; activation of Nrf2/ARE	Alzheimer’s disease	Reduces amyloid plaque formation, decreases tau hyperphosphorylation, and alleviates oxidative stress	[230,231]
EGCG	Inhibits α-synuclein fibrillization; activates PI3K/Akt pathway; reduces mitochondrial dysfunction	Parkinson’s disease	Protects dopaminergic neurons, improves motor function, reduces oxidative burden	[149]
Quercetin	Modulation of mitochondrial biogenesis (via SIRT1 and PGC-1α); anti-inflammatory activity	Alzheimer’s and Parkinson’s diseases	Enhances neuronal energy metabolism and reduces neuroinflammation	[232,233]
Resveratrol	Activation of SIRT1 pathway; mitochondrial protection; anti-aggregatory action	Alzheimer’s and Parkinson’s diseases	Reduces protein misfolding, promotes autophagy, and maintains mitochondrial integrity	[45,234]
Rosmarinic acid	Antioxidant and anti-inflammatory effects; BBB stabilization; inhibition of acetylcholinesterase (AChE)	Alzheimer’s disease	Improves cholinergic neurotransmission, reduces neuroinflammation	[164,235,236]

**Table 7:** Phytochemicals and sleep modulation.

Phytochemicals	Mechanism of Action	Target Pathway/System	Effect on Sleep	References
Naringenin	Enhances melatonin synthesis by upregulating AANAT expression	Melatonin biosynthesis/Circadian rhythm	Improves sleep initiation and rhythm regularity	[239,240]
Luteolin	Stimulates melatonin production and antioxidant defense	Pineal gland/AANAT pathway	Supports sleep cycle alignment and neuroprotection	[241,242]
Resveratrol	Activates SIRT1/PGC-1α, modulates circadian genes	Suprachiasmatic nucleus (SCN)	Improves circadian rhythm regulation and sleep quality	[243,244]
Apigenin	Binds to benzodiazepine site on GABA-A receptor	GABAergic system	Promotes relaxation and sleep without sedation	[245,246]
Valerenic acid derivatives	Allosteric modulation of GABA-A receptors	GABAergic neurotransmission	Reduces sleep latency and increases duration	[247,248]
EGCG	Suppresses pro-inflammatory cytokines via NF-κB inhibition	Neuroinflammation	Improves sleep by reducing inflammation-related disturbances	[249]
Rosmarinic acid	Inhibits TNF-α and IL-6 expression	NF-κB pathway/Inflammatory cytokines	Enhances sleep through anti-inflammatory action	[78,250]

**Table 8:** Phytochemicals in the treatment of attention deficit and executive dysfunction.

Phytochemicals	Mechanism of Action	Target Pathways/Systems	Potential Benefit	References
Salidroside, rosavin (*Rhodiola rosea*)	Enhances dopaminergic transmission	Dopamine signaling in PFC	Improved attention and executive function	[261,262]
Ginsenosides (*Panax ginseng*)	Modulates catecholaminergic neurotransmission; increases receptor sensitivity	Dopamine and norepinephrine systems	Cognitive enhancement, reduced hyperactivity	[260,263]
Quercetin, EGCG	Anti-inflammatory and antioxidant activity; activation of Nrf2 pathway	Neuroinflammation, oxidative stress, microglial activity	Reduced neuroinflammation and improved attention	[108,150]
Bacosides (*Bacopa monnieri*)	Upregulation of BDNF, synaptogenesis	BDNF pathway, cholinergic system	Improved working memory and cognitive processing	[264,265]
Withanolides (*Withania somnifera*)	Neuroprotection and modulation of neurotransmitter systems	Cholinergic and dopaminergic signaling	Enhanced focus and mental clarity	[266]

**Table 9:** Neuroprotective role of phytochemicals in cognitive decline and dementia.

Phytochemicals	Sources	Mechanisms of Action	Relevance to Cognitive Aging	References
Resveratrol	Grapes, red wine	Activation of SIRT1, antioxidant activity, anti-inflammatory	Enhances neuronal survival, delays neurodegeneration, supports cognitive resilience	[271,272]
Curcumin	Turmeric	Inhibition of NF-κB, antioxidant, modulation of amyloid plaque formation	Protects against synaptic loss, improves memory and attention	[210,273]
EGCG	Green tea	Modulation of mitochondrial function, reduces oxidative stress	Improves cognitive performance and brain metabolism in elderly populations	[274,275]
Quercetin	Onions, apples	Scavenges ROS, modulates BDNF, affects epigenetic regulation (e.g., DNA methylation)	Counters neuroinflammation and supports neurogenesis	[276,277]
Hesperidin	Citrus fruits	Enhancement of cerebral blood flow, reduction in neuroinflammatory markers	Improves working memory and reduces cognitive fatigue	[278]
Anthocyanins	Berries	Modulation of Nrf2 pathway, inhibition of neuronal apoptosis	Delays cognitive decline and supports long-term brain health	[279,280]

**Table 10:** Phytochemicals that target early neurodegenerative mechanisms.

Phytochemicals	Natural Sources	Targeted Mechanisms	Potential Neuroprotective Effects	References
Curcumin	Turmeric (*Curcuma longa*)	Inhibition of oxidative stress, NF-κB signaling	Anti-inflammatory, antioxidant, prevents amyloid formation	[288]
Resveratrol	Grapes, red wine, berries	SIRT1 activation, mitochondrial function, antioxidant	Improves neuronal survival, reduces inflammation	[289]
EGCG	Green tea (*Camellia sinensis*)	Modulation of protein aggregation, antioxidative action	Inhibits misfolded proteins, promotes synaptic plasticity	[46,108]
Quercetin	Apples, onions, berries	Antioxidant, acetylcholinesterase inhibition	Protects neurons, enhances cognitive functions	[207]
Huperzine A	Chinese club moss (*Huperzia serrata*)	Acetylcholinesterase inhibition	Enhances memory, protects against neurotoxicity	[221,290]
Ginsenosides	Ginseng (*Panax ginseng*)	Anti-apoptotic, anti-inflammatory, antioxidant properties	Enhances neurogenesis, stabilizes mitochondrial function	[291]
Bacopaside I and II	*Bacopa monnieri*	Cholinergic modulation, antioxidant activity	Improves memory, reduces anxiety	[292]
Withanolides	Ashwagandha (*Withania somnifera*)	Anti-inflammatory, amyloid beta clearance	Reduces plaque burden, supports neuroregeneration	[293]
Luteolin	Celery, green pepper, chamomile	Inhibits microglial activation, antioxidant	Prevents neuroinflammation, supports cognitive health	[30]
Sulforaphane	Broccoli, Brussels sprouts	Nrf2 activation, antioxidant response	Enhances detoxification, reduces oxidative stress	[294]

**Table 11:** Modulation of the gut–brain axis by phytochemicals.

Phytochemicals	Mechanism of Action	Microbiota Effects	Neurological Outcomes	References
Curcumin	Enhancement of gut barrier integrity; inhibition of NF-κB	Increases *Lactobacillus* and *Bifidobacterium*; reduces *Enterobacteriaceae*	Reduces neuroinflammation; improves mood and memory	[305]
Quercetin	Modulation of tight junction proteins; antioxidant activity	Promotes SCFA-producing bacteria	Enhances cognitive performance; reduces anxiety-like behavior	[306]
Rosmarinic acid	Anti-inflammatory effects; regulation of cytokine production	Supports beneficial bacterial growth	Attenuates depressive symptoms; supports emotional regulation	[78,307]
EGCG	Downregulation of pro-inflammatory signaling; antioxidative pathways	Favors microbiota diversity	Mitigates stress-induced behavioral impairments	[148,149,308]
Resveratrol	Activation of SIRT1 and AMPK; improves mitochondrial function	Restores microbiota balance	Supports neurogenesis and cognitive flexibility	[309,310]

**Table 12:** Phytochemicals that affect microbiota–brain interactions.

Phytochemicals	Primary Dietary Sources	Microbiota Modulation	Neuroactive Metabolites or Effects	References
Quercetin	Apples, onions, berries	↑ *Lactobacillus*, *Bifidobacterium*	↑ SCFAs, hesperetin formation (BBB-permeable)	[314]
Curcumin	Turmeric	Modulates gut microbial diversity	Anti-inflammatory, ↑ BDNF expression	[315,316]
EGCG	Green tea	↑ *Bifidobacterium* spp.	Neuroprotection, antioxidant effects	[317]
Hesperidin	Citrus fruits	Biotransformed by *Bacteroides*	↑ Hesperidin (crosses BBB)	[318]
Rosmarinic acid	Herbs (rosemary, basil)	Stabilizes microbiota balance	↓ Neuroinflammation, preserves gut lining	[319]
Resveratrol	Grapes, red wine	↑ *Akkermansia muciniphila*	Modulates synaptic plasticity, anti-depressant effects	[320]

Note: The arrows indicate the direction of change: ↑—increase/enhancement; ↓—decrease/reduction.
